# Critical role of lectin pathway mediated by MBL-associated serine proteases in complement activation for the pathogenesis in systemic lupus erythematosus

**DOI:** 10.1016/j.heliyon.2023.e19072

**Published:** 2023-08-10

**Authors:** Yuko Asanuma, Kazuhisa Nozawa, Masakazu Matsushita, Makio Kusaoi, Yoshiyuki Abe, Ken Yamaji, Naoto Tamura

**Affiliations:** a Department of Internal Medicine and Rheumatology, Juntendo University School of Medicine, Tokyo, Japan; b Department of Internal Medicine and Rheumatology, Juntendo University Koshigaya Hospital, Saitama, Japan

**Keywords:** MASPs, Systemic lupus erythematosus, Lupus nephritis

## Abstract

In complement activation system, although the classical pathway has shown to play a critical role for the pathogenesis of SLE, the role of lectin pathway has remained unknown in the pathogenesis of SLE. As Mannose-binding lectin-associated serine proteases (MASPs) are associated with activation of the lectin pathway, we conducted this study to clarify MASPs associations in the pathogenesis of SLE. We evaluated the serum level of MASPs (MASP-1 and MASP-2) in total 68 SLE patients consisting of 15 patients with biopsy-confirmed membranous lupus nephritis (M-LN), 35 patients with biopsy-confirmed proliferative lupus nephritis (P-LN), and 18 SLE patients without LN (non-LN). Our data showed that the serum levels of MASPs were reduced in both P-LN and non-LN although those of M-LN were not reduced. Our data show that the lectin pathway mediated by MASPs plays a critical role for the pathogenesis of SLE except for M-LN.

## 1 Introduction

Patients with SLE have atypical complement activation originated by immune complexes (ICs), consisting of a variety of autoantigens, such as double stranded DNA (dsDNA) and their antibodies. There are three activation pathways in the complement system: classical, alternative, and lectin pathway. Although the classical pathway is commonly known to play a crucial role in the pathogenesis of SLE, the roles of other pathways still are unknown, especially the lectin pathway. Activation of the lectin pathway is started by recognition of several pattern recognition molecules (PRMs), such as mannose-binding lectin (MBL). The recognition of PRMs is translated into signals by 2 proteases in combination with the PRMs, mannose-binding lectin-associated serine proteases-1 and 2 (MASP-1 and 2). The first step is the autoactivation of MASP-1 and then MASP-1 activates MASP-2. MASP-3 is a splicing variant of the *Masp1* gene, and the *Masp1* gene transcribes another splicing variant Map44, which lacks the serine protease domain [1]. In recent years, the involvement of MASPs has been proven to be important in renal injury, vascular injury, and autoimmune diseases [[2], [3], [4], [5], [6]]. Therefore, the significance of the lectin pathway and the pathogenesis of complement-activated diseases have attracted attention. Recently, there have been some research on MASPs, regarding the pathogenesis of SLE. In a research, lupus-prone MRL/lpr mice with *Masp1*/3 gene knockout had preserved serum C3 levels, less albuminuria, and significantly less glomerular deposition than their wild-type littermates [7]. Troldborg et al. reported that patients with SLE have high serum MASP-1 and MASP-3 levels [8]. Xu et al. reported that serum MASP-2 levels are high in patients with SLE [9]. These findings suggest that MASPs are crucial in the pathology of LN. However, the clinical correlation between the disease activity and serum levels of MASPs has never been reported. Therefore, we measured serum levels of MASP-1/2 and investigated the clinical association of MASPs to the pathogenesis of SLE in this study.

## 2 Materials and methods

### 2.1 Patients and samples

All patients received care at Juntendo University Hospital between March 2012 and March 2020, and met the American College of Rheumatology (ACR) classification criteria for SLE. 68 SLE patients, consisting of 15 patients with biopsy-confirmed membranous lupus nephritis (M-LN), 35 patients with biopsy-confirmed proliferative lupus nephritis (P-LN), and 18 SLE patients without LN (non-LN), were enrolled in this study. Among the SLE patients, 20 patients (P-LN: n = 12, non-LN: n = 8), whom the clinical data before and after the treatment could be obtained, were further evaluated to check serum MASP-1 levels. Normal healthy controls (NHCs) consisted of 12 healthy volunteers, who had no rheumatic disease.

Renal biopsy samples were inspected with light microscopy and direct immunohistochemical staining. Renal histopathology was sorted according to the 2003 revised International Society of Nephrology/Renal Pathology Society (ISN/RPS) criteria. Records on following parameters were obtained: gender, age, history of hypertension and diabetes mellitus, SLE symptoms, prednisolone dose, and use of immunosuppressive agents. Blood samples was obtained for measurement of serum autoantibodies, immune complexes (ICs), serum creatinine (Scr), blood urea nitrogen (BUN), lactate dehydrogenase (LDH), serum albumin, CH50, C3, C4, and estimated glomerular filtration rate (eGFR). The following urine parameters were assessed: urine protein-creatinine ratio (PCR), urine RBC, urine WBC, and urinary casts. Disease activity was evaluated based on the Systemic Lupus Erythematosus Disease Activity Index 2000 (SLEDAI-2K).

### 2.2 Assays of MASP-1 and MASP-2 titers

Serum levels of MASP-1 and MASP-2 were measured using commercially available ELISA kits according to the manufacturer's instructions (Cloud-Clone, Katy, TX).

### 2.3 Statistical analysis

All statistical analyses were performed with EZR [10]. Differences between groups were assessed using the Mann-Whitney *U* test for differences in quantitative parameters, the Fisher's exact test for qualitative parameters, and the Kruskal-Wallis test for differences between three groups. The correlation between serum level of MASP-1 and C3, C4, and CH50 was evaluated by Spearman's rank correlation coefficient. A post hoc power analysis using One-way ANOVA was performed, by G* power (Version 3.1.9.6), to check the appropriateness of the sample size. All tests were two-sided, and the significance level was set at *p* < 0.05.

## 3 Results

### 3.1 Clinical characteristics and laboratory findings of the patients with SLE

As indicated in Table 1, there were no significant differences in demographic and clinical features including fever, mucocutaneous manifestations, musculoskeletal manifestations, serositis, and hematologic manifestations between patients with LN and patients with SLE but no renal manifestations. Significantly lower levels of serum C3, CH50, and albumin were detected in P-LN patients. Regarding the urine data, the proportion of patients with proteinuria, hematuria, pyuria, or urinary casts were significantly higher in P-LN patients.

**Table 1:** Clinical characteristics of patients with membranous LN, proliferative LN, and SLE without LN.

Characteristic	Membranous LN (n = 15)	Proliferative LN (n = 35)	SLE without LN (n = 18)	*p*
Age, median (IQR), years	33.0 [28.5, 44.5]	35.0 [29.5, 44.0]	39.5 [27.3, 55.0]	0.83
Female sex, n (%)	14 (93.3)	29 (82.9)	17 (94.4)	0.36
Hypertension, n (%)	2 (13.3)	6 (17.1)	3 (16.7)	0.94
Diabetes mellitus, n (%)	1 (6.7)	1 (2.9)	2 (11.1)	0.48
**SLE manifestations**				
Fever, n (%)	1 (6.7)	6 (17.1)	4 (22.2)	0.47
**Mucocutaneous**				
Mucosal ulcers, n (%)	0 (0.0)	2 (5.7)	1 (5.6)	0.64
Rash, n (%)	1 (6.7)	11 (31.4)	3 (16.7)	0.13
Alopecia, n (%)	3 (20.0)	2 (5.7)	0 (0.0)	0.08
**Musculoskeletal**				
Arthritis, n (%)	4 (26.7)	5 (14.3)	7 (38.9)	0.13
Myositis, n (%)	1 (6.7)	0 (0.0)	0 (0.0)	0.17
**Serositis**				
Pleurisy, n (%)	1 (6.7)	2 (5.7)	0 (0.0)	0.56
Pericarditis, n (%)	1 (6.7)	1 (2.9)	0 (0.0)	0.53
**Hematologic**				
Thrombocytopenia, n (%)	0 (0.0)	3 (9.7)	2 (11.1)	0.43
Leukopenia, n (%)	0 (0.0)	4 (12.9)	3 (16.7)	0.28
**Serology**				
dsDNA Ab, median (IQR), IU/mL	2.0 [2.0, 13.5]	16.0 [2.0, 125.0]	3.8 [2.0, 14.0]	0.16
C3, median (IQR), mg/dL	82.00 [58.00, 102.00]	63.00 [34.50, 78.00]	98.00 [77.25, 104.00]	**<0.01**
C4, median (IQR), mg/dL	18.00 [12.00, 19.00]	14.00 [6.00, 17.50]	16.00 [11.75, 21.00]	0.22
CH50, median (IQR), mg/dL	32.80 [24.20, 41.00]	25.00 [12.25, 36.90]	36.55 [31.78, 45.30]	**0.02**
IC-C1q, median (IQR), mg/dL	1.50 [1.50, 4.28]	2.15 [1.50, 7.25]	1.50 [1.50, 2.53]	0.2
Albumin, median (IQR), g/dL	3.10 [2.55, 3.60]	3.00 [2.40, 3.35]	3.80 [3.45, 4.10]	**<0.01**
Creatinine, median (IQR), mg/dL	0.64 [0.51, 0.71]	0.64 [0.50, 1.02]	0.55 [0.47, 0.81]	0.5
GFR, median (IQR), mL/min/1.73m^2^	93.70 [73.40, 104.35]	86.90 [49.80, 116.05]	105.05 [64.57, 114.05]	0.66
LDH, median (IQR), U/L	194.00 [173.00, 253.50]	210.00 [168.00, 239.50]	192.50 [167.50, 247.25]	0.9
**Urine**				
Urine PCR, median (IQR), g/24h	1.20 [0.29, 3.38]	1.38 [0.30, 2.73]	0.14 [0.04, 0.23]	**<0.01**
≥5 RBCs/HPF, n (%)	5 (33.3)	21 (60.0)	1 (5.6)	**<0.01**
≥5 WBCs/HPF, n (%)	5 (33.3)	20 (57.1)	0 (0.0)	**<0.01**
Urinary cast, n (%)	8 (53.3)	13 (37.1)	1 (5.6)	**0.01**

LN: Lupus Nephritis, IQR: Inter-Quartile Range, dsDNA: double stranded DNA, PCR: protein-creatinine ratio, HPF: High Power Field, SLEDAI-2K: Systemic Lupus Erythematosus Disease Activity Index 2000.

### 3.2 Serum levels of MASP-1 and MASP-2 in the P-LN, M-LN, and non-LN subsets and normal healthy controls

**Fig. 1:**
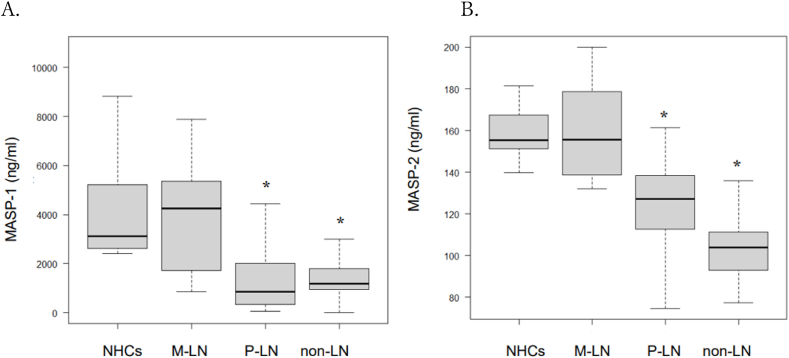
Serum levels of MASP-1 and MASP-2 in healthy controls, patients with MLN, PLN and SLE patients without renal involvement (non-LN) are shown. Significantly lower level of MASP-1 was seen in both P-LN and non-LN, compared to NHCs (Fig. 1A). Similarly, significantly lower level of MASP-2 was seen in both P-LN and non-LN, compared to NHCs (Fig. 1B). Statistical analysis was performed using the Mann-Whitney *U* test, and p-values of less than 0.05 were considered statistically significant. (*).

As shown in Fig. 1A, significantly lower levels of MASP-1 were observed in P-LN patients (median 854.28 ng/mL; IQR 330.60, 2013.23 ng/mL) than in NHCs (median 3112.82 ng/mL; IQR 2640.51, 5185.97 ng/mL). In addition to P-LN patients, the significant reduction of serum MASP-1 was also observed in non-LN patients (median 1190.15 ng/mL: IQR 980.84, 1760.36 ng/mL). Comparable results were seen in MASP-2. The lower levels of MASP-2 were significantly seen in P-LN patients (median 127.20 ng/mL; IQR 112.65, 138.35 ng/mL) than in NHCs (median 155.30 ng/mL; IQR 152.35, 167.10 ng/mL). Significantly lower levels of serum MASP-2 were also observed in non-LN (median 103.85 ng/mL: IQR 94.20, 110.93 ng/mL) (Fig. 1B). The levels of both serum MASP-1 (median 4245.42 ng/mL; IQR 1735.04, 5354.05 ng/mL) and MASP-2 (median 155.70 ng/mL; IQR 138.70, 178.65) in the M-LN patients were comparable to those of NHCs.

### 3.3 Correlation between serum MASP-1 levels and serum levels of C3, C4, CH50, IC-C1q and anti-dsDNA antibodies

**Fig. 2:**
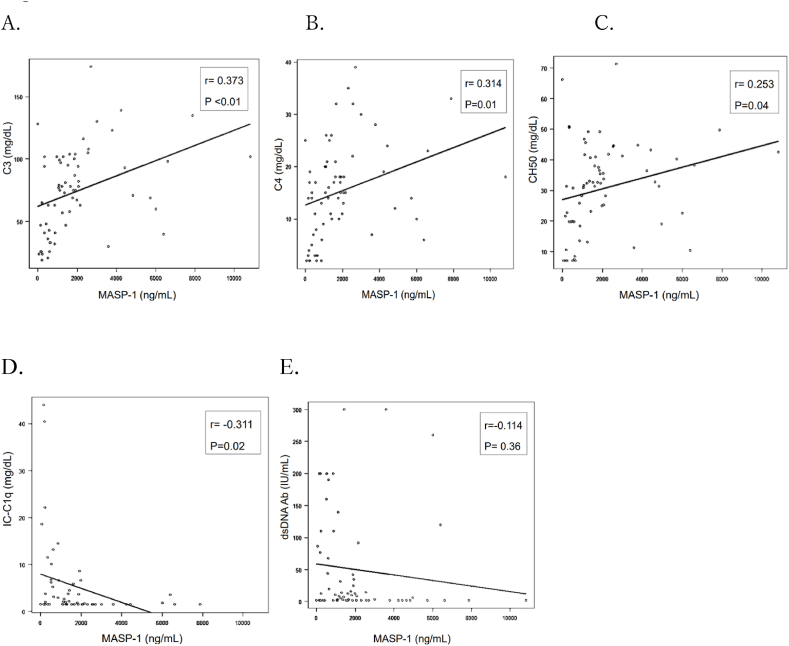
Correlation between serum levels of MASP-1 versus C3, C4, CH50, IC-C1q and anti-dsDNA antibodies is shown. Serum levels of MASP-1 have a positive correlation with serum levels of C3, C4, CH50, and have a negative correlation with serum level of IC-C1q. There was no correlation between serum levels of MASP-1 and anti-dsDNA antibodies. The correlation between serum level of MASP-1 and C3, C4, and CH50 was assessed by Spearman's rank correlation coefficient and p-values of less than 0.05 were considered statistically significant.

The correlation between serum levels of MASP-1 and other serological parameters of SLE disease activity, including C3, C4, CH50 IC-C1q, and anti-ds DNA antibodies, are shown in Fig. 2A–E. Positive correlation between serum levels of MASP-1 and serum C3, C4, and CH50 was observed (Fig. 2A–C). Negative correlation with serum levels of MASP-1 and IC-C1q was observed (Fig. 2D), although no correlation between serum levels of MASP-1 and serum levels of anti-dsDNA antibodies was observed (Fig. 2E).

### 3.4 Comparison of serum MASP-1 levels before and after immunosuppressive treatment

Table 2 shows the clinical characteristics of the SLE patients before and after treatment. Both P-LN patients (n = 12) and non-LN patients (n = 8) had high disease activity of SLE, as SLEDAI-2K median score was 19.5 in P-LN and 14.5 in non-LN, respectively. P-LN patients received prednisolone at a median dose of 47.5 mg/day (IQR 38.4, 50.0) and 10 of the P-LN patients received concomitant immunosuppressants (83.3%), while non-LN patients received prednisolone at a median dose of 32.5 mg/day (IQR 23.8, 37.5) and 7 of the non-LN patients received concomitant immunosuppressants (87.5%). The treatment ameliorated SLE, as several parameters of the disease activity of SLE were improved, including significant reduction of patients with fever and arthritis, restoration of serum levels of C3 and CH50, and decrease of SLEDAI scores (from 19.5 to 4.0 in P-LN, from 14.5 to 4.0 in non-LN), compared with those before treatment.

**Table 2:** Clinical characteristics of patients before versus after treatment.

	SLE with LN (n = 12)		p. value	SLE without LN (n = 8)		p. value
	Before treatment	After treatment		Before treatment	After treatment	
**SLE manifestations**						
Fever, n (%)	5 (41.7)	0 (0.0)	**0.04**	7 (87.5)	0 (0.0)	**<0.01**
Neuropsychiatric, n (%)	1 (8.3)	0 (0.0)	1	4 (50.0)	1 (12.5)	0.28
**Mucocutaneous**						
Mucosal ulcers, n (%)	2 (16.7)	0 (0.0)	0.48	1 (12.5)	0 (0.0)	1
Rash, n (%)	7 (58.3)	0 (0.0)	**<0.01**	4 (50.0)	1 (12.5)	0.28
Alopecia, n (%)	1 (8.3)	1 (8.3)	1	1 (12.5)	2 (25.0)	1
**Musculoskeletal**						
Arthritis, n (%)	7 (58.3)	0 (0.0)	**<0.01**	6 (75.0)	1 (12.5)	**0.04**
Myositis, n (%)	1 (8.3)	0 (0.0)	1	0 (0.0)	0 (0.0)	NA
**Serositis**						
Pleurisy, n (%)	2 (16.7)	0 (0.0)	0.48	1 (12.5)	0 (0.0)	1
Pericarditis, n (%)	1 (8.3)	0 (0.0)	1	0 (0.0)	0 (0.0)	NA
**Hematologic**						
Platelet, median (IQR), x10^4^/μL	13.35 [11.40, 15.70]	23.20 [19.67, 28.73]	**<0.01**	20.80 [17.35, 24.97]	25.15 [21.83, 27.92]	0.14
WBC, median (IQR),/μL	3000 [2475, 4075]	6400 [5275, 8350]	**<0.01**	4350 [2600, 5875]	8050 [5475, 11375]	0.05
**Serology**						
dsDNA Ab, median (IQR), IU/mL	200.0 [150.0, 200.0]	28.0 [15.5, 88.8]	**0.01**	48.5 [24.3, 88.5]	7.7 [2.0, 50.8]	0.09
C3, median (IQR), mg/dL	25.00 [20.50, 30.75]	41.50 [33.00, 68.50]	**<0.01**	48.00 [41.25, 55.50]	85.00 [73.75, 88.75]	**0.01**
C4, median (IQR), mg/dL	3.00 [2.75, 8.75]	10.00 [6.75, 14.00]	0.05	8.50 [5.00, 12.75]	15.00 [10.00, 20.50]	0.13
CH50, median (IQR), mg/dL	7.00 [7.00, 10.55]	17.10 [14.00, 22.35]	**<0.01**	23.95 [9.90, 28.90]	38.25 [25.40, 43.53]	0.05
IC-C1q, median (IQR), mg/dL	8.65 [4.22, 19.50]	2.75 [1.80, 4.85]	0.05	1.95 [1.50, 5.50]	1.50 [1.50, 2.00]	0.41
Albumin, median (IQR), g/dL	3.10 [2.28, 3.40]	3.35 [2.75, 3.85]	0.28	3.40 [3.05, 3.70]	3.65 [3.08, 3.95]	0.29
Creatinine, median (IQR), mg/dL	0.74 [0.66, 0.96]	0.68 [0.56, 0.77]	0.23	0.48 [0.42, 0.52]	0.46 [0.41, 0.49]	0.75
GFR, median (IQR), mL/min/1.73m^2^	79.15 [61.70, 94.22]	97.00 [79.90, 109.90]	0.23	127.70 [111.27, 135.62]	129.45 [115.15, 156.55]	0.60
LDH, median (IQR), U/L	261.50 [239.00, 361.25]	206.00 [182.25, 222.50]	**<0.01**	372.00 [274.50, 651.75]	221.00 [186.75, 262.75]	**0.02**
**Nephrology**						
Urine PCR, median (IQR), g/24h	1.19 [0.66, 1.53]	0.36 [0.10, 1.45]	0.11	0.16 [0.03, 0.24]	0.07 [0.03, 0.09]	0.20
≥5 RBCs/HPF, n (%)	5 (41.7)	2 (16.7)	0.37	0 (0.0)	0 (0.0)	NA
≥5 WBCs/HPF, n (%)	5 (41.7)	0 (0.0)	**0.04**	0 (0.0)	0 (0.0)	NA
Urinary cast, n (%)	7 (58.3)	2 (16.7)	0.09	0 (0.0)	0 (0.0)	NA
**SLEDAI-2K, median (IQR)**	19.50 [17.75, 22.00]	4.00 [4.00, 10.50]	**<0.01**	14.50 [11.00, 16.00]	4.00 [2.00, 4.50]	**<0.01**

SLE: Systemic Lupus Erythematosus, LN: Lupus Nephritis, IQR: Inter-Quartile Range, dsDNA: double stranded DNA, PCR: protein-creatinine ratio, HPF: High Power Field, SLEDAI-2K: Systemic Lupus Erythematosus Disease Activity Index 2000.

**Fig. 3:**
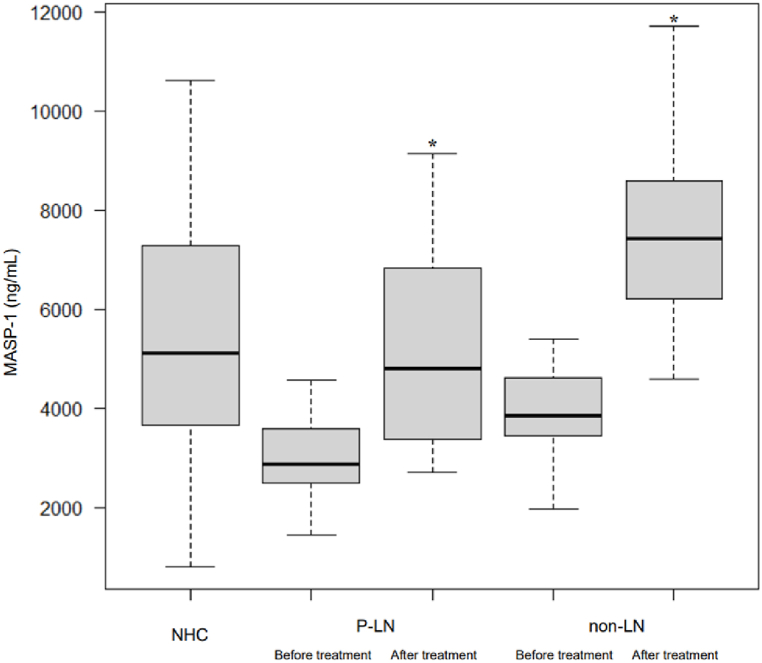
Serum levels of MASP-1 in healthy controls, patients with PLN and SLE patients without renal involvement (non-LN), before and after treatment, are shown. The levels of serum MASP-1 were significantly restored after treatment in both P-LN and non-LN, compared to those before treatment, respectively. Statistical analysis was performed using the Mann-Whitney *U* test, and p-values of less than 0.05 were considered statistically significant. (*).

Fig. 3 shows serum MASP-1 levels of the SLE patients before and after the treatment. Serum levels of MASP-1 before the treatment were significantly reduced in both P-LN patients (median 2865.30 ng/mL; IQR 2644.10, 3556.59 ng/mL) and non-LN patients (median 3836.70 ng/mL; IQR 3545.45, 4238.90 ng/mL) compared to NHCs (median 5111.26 ng/mL; IQR 3664.48, 7269.76 ng/mL). The reduction of MASP-1 was restored after treatment, concomitant with the disease amelioration in P-LN (median 4807.96 ng/mL; IQR 3407.97, 6802.36 ng/mL) similar to non-LN (median 7423.08 ng/mL; IQR 6672.57, 8107.82 ng/mL) compared with the level before the treatment.

### 3.5 Sample size

A total of 68 SLE patients enrolled in the present study comprising of 15 M-LN, 35 p-LN and 18 non-LN. We performed a post-hoc power nalysis with an effect size of 0.2, an alpha error prob of 5%, and total sample size of 68. The power was calculated as 0.283.

## 4 Discussion

In this study, we discovered that serum levels of both MASP-1 and MASP-2 were significantly reduced in P-LN and non-LN compared to NHCs, although M-LN was similar to the level of NHCs (Fig. 1A and B). We also found that the decrease of serum MASP-1 level in P-LN was reversed after treatment, parallel with the disease's amelioration. A similar result was also observed in non-LN patients (Fig. 3, Table 2). Moreover, the serum level of MASP-1 was positively correlated with those of CH50, C3, and C4, and negatively correlated with those of ICs (Fig. 2).

In SLE, the classical pathway can be activated by autoantibody or immune complexes (ICs) consisting of autoantibodies and autoantigens. As presence of serum autoantibodies is one of the characteristics of SLE, the abnormal complement activation of the classical pathway is considered to be crucial in the pathogenesis of SLE. In contrast to the classical pathway, the lectin pathway is mainly believed to be engaged in the innate defense and is triggered by the binding of MBL to one of its ligands [11]. Panda et al. reported that plasma MBL levels were significantly higher in SLE patients compared to NHCs and correlated with the SLEDAI score. Moreover, higher and intermediate MBL levels had significantly high association to LN [12]. Regarding MASPs' association in the pathogenesis of SLE, only two studies have been reported. Machida et al. reported that *MASP-1* gene knock out MRL/*lpr* mice, that lack MASP-1/3, had significantly less glomerular C3 deposition and lower glomerular pathological score [7]. This suggests that MASP-1/3 has a significant part in the development of lupus-like glomerulonephritis in MRL/*lpr* mice by the lectin pathway. Xu et al. demonstrated significantly higher expression of serum MASP-2 in patients with SLE along with *MASP-2* gene polymorphism, compared to NHCs and other rheumatic diseases including rheumatoid arthritis, osteoarthritis, Sjogren's syndrome, and ankylosing spondylitis. This indicated that MASP-2 may be associated with SLE pathogenesis and may be a potential diagnostic biomarker of SLE [9]. However, our study demonstrated that both serum MASP-1 and MASP-2 levels decreased in active SLE, compared to NHCs (Fig. 1A and B), contradicting previous reports, and that the decrease of MASP-1 was normalized after treatment (Fig. 3). In the pathogenesis of SLE, the lectin pathway mediated by MASPs may be associated with the disease progression and MASPs may be excessively consumed during the development of SLE, subsequently resulting in the decrease of serum levels of MASPs, like the mechanisms of hypocomplementemia in SLE. Furthermore, we demonstrated that the serum level of MASP-1 was positively correlated with those of CH50, C3, and C4, and negatively correlated with those of ICs (Fig. 2). Notably, it has been reported that low complement levels have not always proved effective as a disease activity marker in SLE, especially in patients without nephritis [13]. Therefore, MASPs may be an alternative biomarker for the disease activity of SLE (except for patients with M-LN), as our data demonstrated that the decrease of serum MASP-1 level was reversed after treatment (Fig. 3, Table 2).

Systemic lupus erythematosus (SLE) is an autoimmune disease that harms multiple organ systems. Among all organ damages, lupus nephritis (LN) is one of the most serious complications of SLE. According to the International Society of Nephrology/Renal Pathology 2003 classification, LN is divided into 6 classes by glomerular pathology. Amongst these classes, proliferative nephritis (class III/IV) and membranous nephritis (class V) have the capacity to cause lasting damage.

It has been reported that class III/IV P-LN are highly inflammatory, while class V M-LN is less inflammatory [14]. The complement system has been uncultivated in membranous nephropathy (MN) including idiopathic MN and secondary MN, such as M-LN. In idiopathic MN, IgG4 has been reported as the dominant subclass, and because IgG4 does not bind C1q, most idiopathic MN cases show weak C1q deposits. Therefore, in M-LN, the complement system may not be as important for the pathogenesis of SLE compared to P-LN. That may be the reason the serum MASPs levels of patients with M-LN were not reduced in the present study. Since renal biopsy may not be an option in some cases, it is important to find non-histological factors that is useful to distinguish P-LN and M-LN, because their treatment protocol and renal prognosis vary. Although the types of LN are clinically important, clinical factors predictive of both P-LN and M-LN are limited. Although, prevalence of autoantibodies, including anti-dsDNA and C1q, has been researched in SLE to determine their value as a marker of nephritis, their association to LN histology remains controversial. Kwon et al. reported that anti-ds DNA antibodies had a high sensitivity in discriminating P-LN from M-LN [15]. Though we tried to evaluate anti-ds DNA antibodies as a biomarker discriminating P-LN from M-LN in the present study, statistical differences of the prevalence of anti-ds DNA antibodies were not observed in comparison with P-LN and M-LN (data not shown). Regarding M-LN, although several biomarkers, such as M-type phospholipase A2 receptor, have been reported in idiopathic membranous nephropathy [16], no useful biomarker for diagnosis of M-LN has been reported so far. Our data demonstrated that serum levels of MASPs differ between P-LN and M-LN, so MASPs may be used for a biomarker discriminating P-LN from M-LN, as well as evaluating the disease activity of SLE. To the best of our knowledge, this is the first study to evaluate the potential use of MASPs as biomarkers of pathological parameters in SLE.

There are some limitations to this study. The sample size was small, which might limit the applicability of serum MASPs: The test power of this study was 0.283, which implies that the sample size may be insufficient, and significant differences may have been detected where they should not. In order to keep the test power over 0.8, we needed a total of 246 patients, so we plan to assess a new set of patients from our ongoing prospective multi-center study as soon as sufficient number of data is collected. Because of the retrospective nature of our study, there might be limitations in determining variables that might influence the results.

In conclusion, the lectin pathway of complement activation mediated by MASPs may play a critical role in the SLE pathogenesis, except for M-LN. The monitoring of serum MASPs is useful for not only evaluation of the disease activity, but in distinguishing P-LN from M-LN.

## Declarations

### Author contribution statement

Yuko Asanuma: Conceived and designed the experiments; Performed the experiments; Analyzed and interpreted the data; Contributed reagents, materials, analysis tools or data; Wrote the paper.

Kazuhisa Nozawa, M.D., Ph.D.: Conceived and designed the experiments; Analyzed and interpreted the data; Contributed reagents, materials, analysis tools or data; Wrote the paper.

Masakazu Matsushita, M.D., Ph.DORCID; Makio Kusaoi, M.D., Ph.DORCID; Yoshiyuki Abe, M.D., Ph.D; Ken Yamaji, M.D., Ph.D; Naoto Tamura, M.D., Ph.D.: Analyzed and interpreted the data.

### Data availability statement

Data included in article/supp. material/referenced in article.

### Ethics statement

This study was conducted in accordance with the Declaration of Helsinki and the Ethics Guidelines for Clinical Research issued by Japan's Ministry of Health, Labor and Welfare. The ethics committee of Juntendo University Hospital approved this study (approval number: 20-017). Informed consent was obtained in the form of opt-out on the hospital's website (https://www.gcprec.juntendo.ac.jp/kenkyu/).

## Declaration of competing interest

The authors declare that they have no known competing financial interests or personal relationships that could have appeared to influence the work reported in this paper.
